# Bone Morphogenetic Protein 7 Effect on Human Glioblastoma Cell Transmigration and Migration

**DOI:** 10.3390/life11070708

**Published:** 2021-07-17

**Authors:** Ting-Chung Wang, Sheng-Jie Luo, Shun-Fu Chang

**Affiliations:** 1 Department of Neurosurgery, Chiayi Chang Gung Memorial Hospital, Chiayi 613, Taiwan; northernblotting@gmail.com (T.-C.W.); shengluo2020@gmail.com (S.-J.L.); 2 School of Medicine, College of Medicine, Chang Gung University, Taoyuan 333, Taiwan; 3 Graduate Institute of Clinical Medical Sciences, College of Medicine, Chang Gung University, Taoyuan 333, Taiwan; 4 Department of Medical Research and Development, Chiayi Chang Gung Memorial Hospital, Chiayi 613, Taiwan

**Keywords:** bone morphogenetic protein 7, glioblastoma, p75 neurotrophin receptor, Smad5

## Abstract

Glioblastoma, World Health Organization—grade IV, is the most malignant glioma type and it is still an incurable tumor due to the high level of heterogeneity and uncontrolled metastatic nature. In addition to the tumorigenicity-suppressing activity, bone morphogenetic protein 7 (BMP7) has recently been found for its invasion-promoting role in glioblastoma. However, the detailed and precise mechanism in this issue should have more elucidation. Thus, in this study, we determined the BMP7 effect on glioblastoma transmigration and migration regulations and the underlying mechanisms. Human LN18/LN229 glioblastoma cells were used in this study. Our results showed a higher BMP7/pSmad5 level in human malignant glioma tissues compared to healthy brain tissues. In addition, it was demonstrated that endogenous and exogenous BMP7 stimulation could increase the transmigration and migration capabilities of human LN18/LN229 glioblastoma cells. Moreover, this event is regulated by Smad5 and p75 neurotrophin receptor (p75NTR) signaling. Furthermore, unexpected data are that the Smad1 gene knockdown could lead to the cell death of human LN18 glioblastoma cells. Overall, the present study finds that the invasion-promoting activity of BMP7 might be an autocrine stimulation of glioblastoma and this effect could be regulated by Smad5-p75NTR signaling.

## 1. Introduction

Glioma is still a most malignant tumor of brain due to its extremely high invasion and diffusion properties [1,2,3]. Moreover, a very high percentage of recurred sites of glioma have been found to appear within 2.5 cm of the primary glioma resection margin [4]. According to its aggressiveness, glioma has been classified into four grades. Glioblastoma, the grade IV level, is the most malignancy found and has still been indicated as an incurable disease. Patients with glioblastoma have only 12~15-month survival days after the final diagnosis, even if they are treated using the most aggressive clinical therapy, including the combination of surgery, chemotherapy with temozolomide, and radiotherapy [1,2,3]. The major barriers that limit the treating-efficiency improvement for glioblastomas are their high level of heterogeneity, including the presence of glioma initiating cells (GICs), and uncontrolled metastatic nature [1,3,5,6]. Thus, further examining and understanding the glioblastomas’ genetic variations and migration/invasion mechanisms, as well as screening new genetic and molecular targets are becoming imperative to provide an effective improvement of its low survival rate.

The bone morphogenetic protein (BMP) family and its specific downstream signaling, including receptors and Smad1/5 pathway, have been considered as the context-dependent modulators due to their multiple activity in almost all types of tissues and since they could play either a positive or negative role in regulating the pathogenesis, including the tumor development [7,8]. In gliomas, BMP2 and BMP4 have been proposed to be a potential predictor for the prognosis of patients [9,10]. BMP4 is the most well-studied BMP member in gliomas, including in vivo and in vitro researches, and has been evidenced against the gliomas’ tumorigenicity through inducing their differentiation into astrocytes and neuronal/oligodendroglia cells [11]. Therefore, BMP4 has been indicated as a potential anti-glioma drug candidate. In most of the studies, BMP7 has also been elucidated an anti-proliferation and anti-survival role in glioma by its growth inhibitory and differentiation inducer activity [12,13,14,15]. However, recently, it has been further indicated that BMP7 has another potential capability that could block the glioblastoma invasion through the Snail pathway [13]. Thus, the dual regulatory role of BMP7 in glioma that dissociates the invasion capability from tumorigenicity has been proposed, but their detailed and precise mechanism in glioma development needs to be further explored and examined.

The p75 neurotrophin receptor (p75NTR), a member of the tumor necrosis factor receptor family, is a multiple function receptor, which could affect different cell fates (proliferation, apoptosis, and survival), while associating with a different receptor (Trk or Sortilin) or acting alone under nerve growth factor (NGF) or neurotrophin stimulations [16,17,18,19]. In glioblastoma researches, a high level of p75NTR has been found in over 85% of the age of clinical cases and in vitro and in vivo studies. It has also been indicated that membrane p75NTR could be cleaved by γ-secretase and subsequently stimulate the glioblastoma cell migration and invasion capability [1,20]. Accumulating data further find that the γ-secretase activity inhibition significantly attenuates the metastasis of high-p75NTR-expression glioblastoma [20,21]. Thus, p75NTR and γ-secretase have been indicated as a great pharmaceutic target candidate for glioblastoma therapy.

In this study, we investigated the potential role of BMP7 in glioblastoma cell transmigration and migration capabilities. We found a higher BMP7/pSmad5 level in clinical malignant glioma and found an autocrine effect of BMP7 on glioblastoma cell transmigration and migration. In this BMP7 event, we further demonstrated that p75NTR could be upregulated in glioblastoma cells by Smad5 signaling. Moreover, we also had a surprising Smad1 data in glioblastoma cell survival. Our findings provide new insights into the understanding and elucidation of autocrine role of BMP7 and Smad1/5 signaling in promoting the glioblastoma metastasis.

## 2. Materials and Methods

### 2.1. Materials

BMP7-specific antibody (sc-517294) and IgG negative control antibody (sc-2025) were purchased from Santa Cruz (Dallas, TX, USA). Smad1/5/9-specific antibody (ab80255) was purchased from Abcam (Cambridge, MA, USA). p75NTR-specific antibody (GTX61425) was purchased from GeneTex (Irvine, CA, USA), while phospho-Smad1/5- (#9516) and β-actin-specific (#3700) antibodies were purchased from Cell Signaling Technology (Beverly, MA, USA). Biotinylated secondary antibody was purchased from Biocompare (BP-9200, South San Francisco, CA, USA). In addition, control (4390844)-, BMP7 (S2035)-, p75NTR (S194655)-, Smad1 (HSS106248)-, and Smad5 (HSS106259)-specific siRNAs were purchased from Thermo (Waltham, MA, USA). Recombinant human BMP7 (BMP7) was purchased from Sigma-Aldrich (St Louis, MO, USA). All the other chemicals of reagent grade were obtained from Sigma-Aldrich (St Louis, MO, USA).

### 2.2. Tissue Microarray and Immunohistochemical Stain

The commercial human brain tumor tissue microarray was purchased from US Biomax (GL807, Rockville, MD, USA) and was used to analyze the BMP7 level by immunohistochemical stain. In brief, the slides were incubated with the BMP7-specific primary antibody, biotinylated secondary antibody, and a labeling solution. Then, the slides were counterstained with Hematoxylin solution and analyzed [22]. BMP7 immunostaining was determined independently by two well-trained investigators according to a modified score. Briefly, the scores were determined by the extent of positive staining: 0 = 0%; 1 = 1–50%; 2 = 50–100% [23]. The score was then summed and divided by the number of samples.

### 2.3. Cell Culture

Human LN18 and LN229 glioblastoma cells were purchased from the cell bank (American Type Culture Collection, Manassas, VA, USA). Both of the cells were cultured in Dulbecco’s modified Eagle’s medium (DMEM) supplemented with 10% fetal bovine serum (FBS) and 1% Penicillin-Streptomycin (P/S) and incubated in a 37 °C incubator supplemented with 5% CO_2_. DMEM medium, FBS, and P/S were purchased from Thermo (Waltham, MA, USA).

### 2.4. Transmigration Assay

The transmigration assay was performed in a transwell chamber with 8 μm pores (BD Biosciences, Bedford, MA, USA) and cell culture plate according to the manufacturer’s protocols. In brief, cells were cultured in the upper wells and allowed to transmigrate through the membrane after stimulation. After 24 h, the cells on the lower surface of the chamber were fixed using methanol and then stained using 1% Crystal violet. The stained cells were photographed in the microscope and counted.

### 2.5. Migration (Wound-Healing) Assay

The migration assay was performed by the wound-healing experiment using the two-well culture-insert (Ibidi, Grafelfing, Bavaria, Germany). In brief, cells were cultured in both wells of culture-insert. After stimulation, the culture-inserts were removed to allow cell migration. After 24 h, the migration levels were photographed in the microscope and images were quantified.

### 2.6. MTT Assay

Cells were transfected with control- or BMP7-specifc siRNA. After transfection and further incubation with the indicated time, the culture medium was changed with DMEM containing 0.02% 3-(4.5-dimethylthiazol-2-yl)-2,5- diphenyltetrazolium bromide (MTT, Sigma-Aldrich, St. Louis, MO, USA), and then further incubated for 4 h. Finally, the medium was replaced with 200 μL of DMSO and the absorbance was read at 570 nm by the DTX880 Multimode Detector (Beckman Coulter, Brea, CA, USA). All the experiments were performed in triplicates.

### 2.7. Western Blot Analysis

After collection, the cells were lysed in a cell lysis buffer (#9803, Cell Signaling Technology, Beverly, MA, USA) mixed with protease inhibitor cocktail (Roche, Indianapolis, IN, USA). The cell lysates were quantified and the equal concentration of proteins were loaded and analyzed in 10% SDS-PAGE. The separated proteins were transferred onto a nitrocellulose blotting membrane (Millipore, Billerica, MA, USA). Then, the membrane was blocked with non-fat milk and probed with the indicated primary antibodies and secondary antibodies. The blotting bands were detected using the Western-Light chemiluminescent detection system (Applied Biosystems, Foster, CA, USA).

### 2.8. The siRNA Transfection

After culture overnight (DMEM with 10% FBS but no P/S), the cells (~80% cell density) were transfected with the designed commercial siRNAs, including control, BMP7, p75NTR, Smad1 or Smad5, using the Lipofactamine RNAiMAX Reagent (Thermo, Waltham, MA, USA).

### 2.9. Statistical Analysis

The quantified data from the transmigration, migration, and Western blot results (three independent experiments) were expressed as the mean ± SD. Statistical analyses were measured by the SPSS statistical software package (SPSS/PC+, SPSS Inc., Chicago, IL, USA). Inter-group comparisons of continuous variables were performed by the Student’s test or one-way analysis of variance (ANOVA). A *p*-value < 0.05 was indicated as significant.

## 3. Results

### 3.1. A Higher BMP7-pSmad5 Level in Human Malignant Glioma

**Figure 1:**
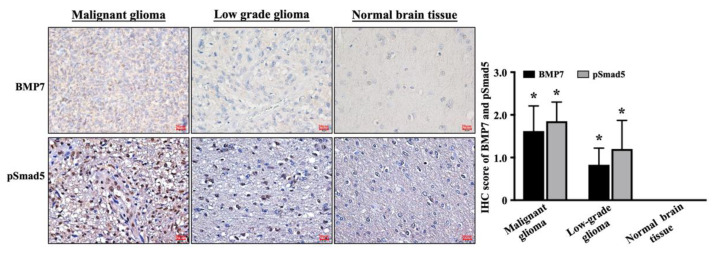
A higher BMP7-pSmad5 level in human malignant glioma. BMP7 and pSmad5 levels in malignant glioma, low-grade glioma, and normal brain tissue were analyzed by immunohistochemical stain with BMP7- and pSmad5-specific antibodies. The mean immunohistochemical stain scores of BMP7 and pSmad5 levels were shown in the right panel. Results are representative of three independent experiments with similar results. Scale bars are 20 μm. *, *p* = 0.001 vs. normal brain tissue.

To identify the exact role of BMP7 and its downstream Smad5 signaling in glioma development, a commercial brain tumor tissue microarray (GL807, US Biomax Inc., Rockville, MD, USA), which included thirty-three astrocytic tumor tissues and five healthy brain tissues, was used and an immunohistochemical stain was performed using the BMP7- and pSmad5-speific antibodies. Tumor grade data were confirmed by US Biomax Inc. Three specimens were grade I, nine specimens were grade II, 12 specimens were grade III, and nine specimens were grade IV. A tendency of higher BMP7 (upper panel in Figure 1)/pSmad5 (down panel in Figure 1) level was shown in the malignant glioma tissues. However, the BMP7 and pSmad5 levels were rarely found in healthy brain tissue. Moreover, staining with an IgG negative control antibody showed the BMP7 antibody specificity in grade 4 glioblastoma tissue (Figure S1). The mean immunohistochemical stain scores of BMP7 and pSmad5 levels were 1.62 ± 0.59 and 1.85 ± 0.45, respectively, in malignant tumor (including Grade III and IV), which was significantly higher than that in low-grade tumor (including Grade I and II) (0.83 ± 0.39 and 1.20 ± 0.67, respectively) and normal brain tissue BMP7 immunoreactivity (*p* = 0.001) (right panel in Figure 1).

### 3.2. Endogenous BMP7 Affects Human LN18/LN229 Glioblastoma Cell Transmigration and Migration

**Figure 2:**
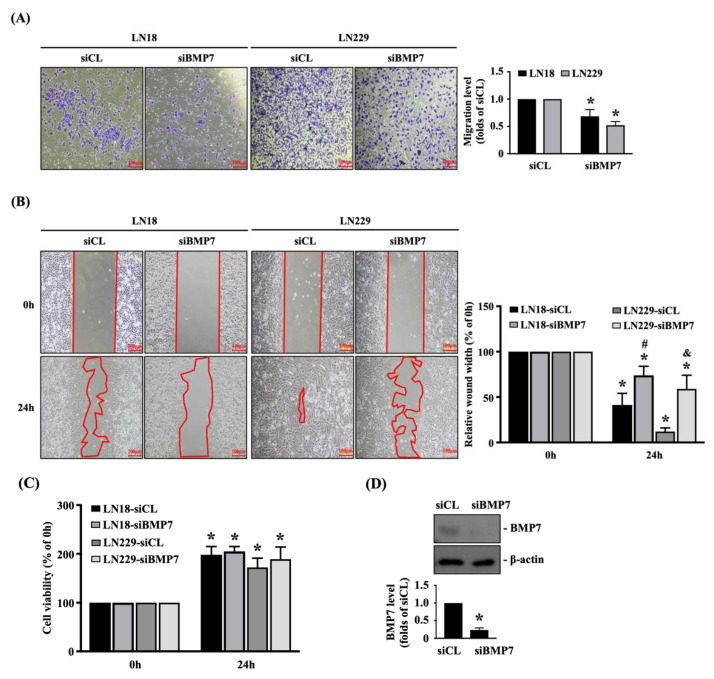
Endogenous BMP7 affects human LN18/LN229 glioblastoma cell transmigration and migration. Human LN18 and LN229 glioblastoma cells were transfected with control- or BMP7-specific siRNA and incubated for 48 h and then the (**A**) cell transmigration (transwell assay) and (**B**) migration capabilities (wound-healing assay), (**C**) cell viability (MTT assay), and (**D**) BMP7 expression (Western blot) were determined. Results in (**A**–**D**) are representative of three independent experiments with similar results. Statistic data in (**A**–**D**) are mean ± SEM from three independent experiments. (**A**–**D**) *, *p* < 0.05 vs. siCL group (**A**,**D**) and siCL-0 h/siBMP7-0 h group (**B**,**C**). (**B**) #, *p* < 0.05 vs. siCL-24 h group in LN18 cells; &, *p* < 0.05 vs. siCL-24 h group in LN229 cells. Scale bars in (**A**,**B**) are 100 μm.

To further elucidate the role of endogenous BMP7 upregulation in glioma malignancy, human LN18 and LN229 glioblastoma cells were transfected with control- or BMP7-specific siRNA and incubated for 48 h and then the transmigration and migration capabilities of both glioblastoma cells were determined by the transwell and wound-healing assay, respectively. It was also shown that the BMP7 gene knockdown could significantly decrease the transmigration level (Figure 2A) and migration level (Figure 2B) of human LN18 and LN229 glioblastoma cells compared to the control-specific siRNA-transfected cells. In order to further clarify the decreased transmigration and migration levels induced by BMP7 gene knockdown were not because of the cell survival inhibition, the viability of both glioblastoma cells was determined by the MTT assay. It was also shown that the BMP7 gene knockdown does not affect the cell viability of both glioblastoma cells (Figure 2C). The knockdown efficiency of BMP7-specific siRNA was examined in human LN18 glioblastoma cells, which resulted in ~70% reduction in the endogenous BMP7 expression level (Figure 2D and Figure S2).

### 3.3. Exogenous Recombinant Human BMP7 Promotes Human LN18 Glioblastoma Cell Transmigration and Migration

**Figure 3:**
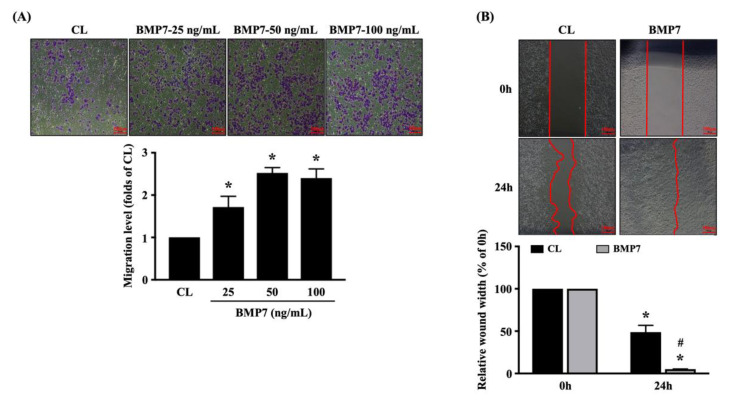
Exogenous recombinant human BMP7 promotes human LN18 glioblastoma cell transmigration and migration**.** (**A**) Human LN18 glioblastoma cells were kept as the controls or treated with BMP7 (25, 50, and 100 ng/mL) for 24 h and then the transmigration capability of cells was determined by the transwell assay. (**B**) Human LN18 glioblastoma cells were kept as the controls or treated with 50 ng/mL BMP7 for 0 and 24 h and then the migration capability was determined by the wound-healing assay. Results in (**A**,**B**) are representative of three independent experiments with similar results. Statistic data in (**A**,**B**) are mean ± SEM from three independent experiments. (**A**,**B**) *, *p* < 0.05 vs. CL group (**A**) and CL-0 h group (**B**). (**B**) #, *p* < 0.05 vs. CL-24 h group. Scale bars in (**A**,**B**) are 100 μm.

Human LN18 glioblastoma cells were kept as the controls or treated with BMP7 (25, 50, and 100 ng/mL) for 24 h and then the transmigration capability of cells was determined by the transwell assay. BMP7 at 25 and 50 ng/mL significantly increased the transmigration level of human LN18 glioblastoma cells in a dose-dependent manner compared to the untreated controls (Figure 3A). The increase in human LN18 glioblastoma cell transmigration level of 100 ng/mL BMP7 stimulation slightly declined compared to the 50 ng/mL BMP7-treated cells, but it still higher than the untreated controls (Figure 3A). Cells were kept as the controls or treated with 50 ng/mL BMP7 for 0 and 24 h and then the migration capability was determined by the wound-healing assay. It was shown that BMP7 could accelerate the human LN18 glioblastoma cell migration after 24 h of treatment (Figure 3B).

### 3.4. BMP7-Smad5 Increases p75NTR Expression in Human LN18 Glioblastoma Cells

**Figure 4:**
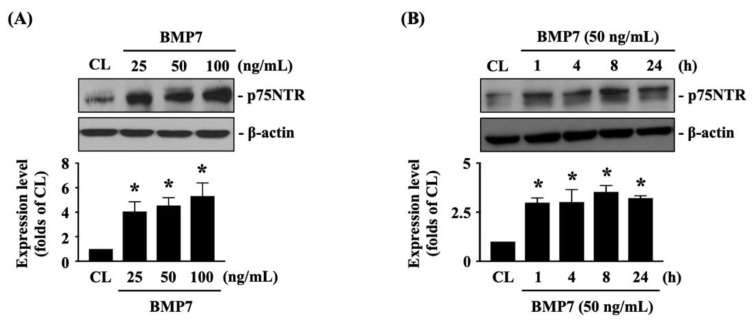
BMP7 increases p75NTR expression in human LN18 glioblastoma cells. (**A**,**B**) Human LN18 glioblastoma cells were kept as the controls or treated with BMP7 (25, 50, 100 ng/mL) for 8 h (**A**) or treated BMP7 (50 ng/mL) for 1, 4, 8, and 24 h (**B**) and then the p75NTR protein expression was determined by Western blot. Results in (**A**,**B**) are representative of three independent experiments with similar results. Statistic data in (**A**,**B**) are mean ± SEM from three independent experiments. *, *p* < 0.05 vs. CL group.

**Figure 5:**
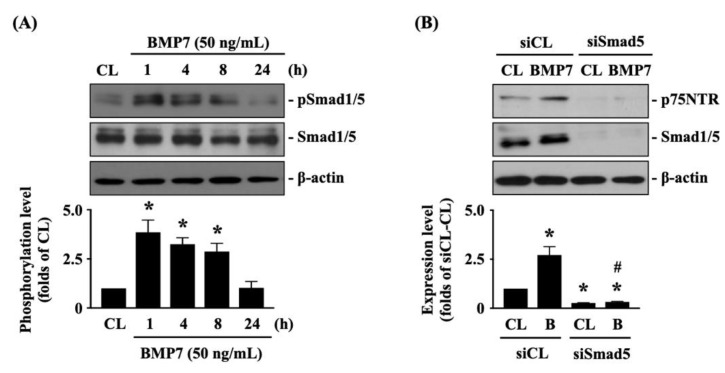
Smad5 regulates p75NTR expression in BMP7-stimulated human LN18 glioblastoma cells. (**A**) Human LN18 glioblastoma cells were kept as the controls or treated with BMP7 (50 ng/mL) for 1, 4, 8, and 24 h and then the Smad1/5 phosphorylation was determined by Western blot. (**B**) Human LN18 glioblastoma cells were transfected with control-, Smad1- or Smad5-specific siRNA, kept as the controls or treated with BMP7 (50 ng/mL) for 24 h and then the p75NTR protein expressions were determined by Western blot. Results in (**A**,**B**) are representative of three independent experiments with similar results. Statistic data in (**A**,**B**) are mean ± SEM from three independent experiments. (**A**) *, *p* < 0.05 vs. CL group. (**B**) *, *p* < 0.05 vs. siCL-CL group; #, *p* < 0.05 vs. siSmad5-CL group.

The p75NTR is important for glioma cell metastasis [1]. Next, we determined whether p75NTR plays a role in BMP7-promoted human LN18 glioblastoma cell transmigration and migration. Cells were kept as the controls or treated with BMP7 (25, 50, 100 ng/mL) for 8 h or treated with BMP7 (50 ng/mL) for 1, 4, 8, and 24 h and then the p75NTR protein expression was determined by Western blot. It was shown that BMP7 increases the p75NTR protein expression in human LN18 glioblastoma cells in a dose-dependent manner compared to the untreated controls (Figure 4A and Figure S2). Moreover, p75NTR protein expression was rapidly induced in 1 h as well as a sustained upregulation in 24 h after BMP7 (50 ng/mL) stimulation (Figure 4B and Figure S2). Smad1/5 signaling is the BMP7-specific downstream pathway [7]. Thus, cells were further kept as the controls or treated with BMP7 (50 ng/mL) for 1, 4, 8, and 24 h and then the Smad1/5 phosphorylation was determined by Western blot. BMP7 significantly induced an increase in Smad1/5 phosphorylation within 1 h and then this induction declined but still kept elevated after 8 h (but not 24 h) of treatment (Figure 5A and Figure S2). Cells were transfected with control-, Smad1- or Smad5-specific siRNA and then kept as the controls or treated with BMP7 (50 ng/mL) for 24 h. The p75NTR protein expression in human LN18 glioblastoma cells was determined by Western blot. It was shown that the Smad5 gene knockdown significantly blocks BMP7-increased p75NTR protein expression in human LN18 glioblastoma cells (Figure 5B and Figure S2). Moreover, Smad5-specific siRNA could effectively attenuate the endogenous Smad5 expression level of human LN18 glioblastoma cells (Figure 5B and Figure S2). Surprisingly, Smad1 gene knockdown in human LN18 glioblastoma cells seriously resulted in their cell death. Thus, we cannot identify the Smad1 role in p75NTR upregulation of BMP7 stimulation in human LN18 glioblastoma cells.

### 3.5. Smad5-p75NTR Signaling Regulates BMP7-Promoted Transmigration and Migration of Human LN18 Glioblastoma Cells

**Figure 6:**
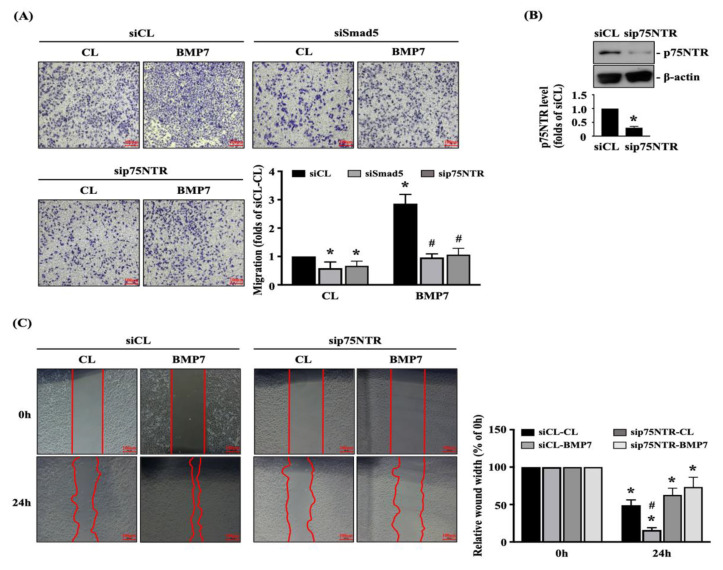
Smad5-p75NTR signaling regulates BMP7-promoted human LN18 glioblastoma cell transmigration and migration. Human LN18 glioblastoma cells were transfected with control-, Smad5- or p75NTR-specific siRNA and then kept as the controls or treated with BMP7 (50 ng/mL) for 24 h. (**A**–**C**) The transmigration capabilities (**A**), p75NTR expression (**B**), and migration capabilities (**C**) of human LN18 glioblastoma cells were determined by the transwell assay, Western blot, and wound-healing assay, respectively. (**A**) *, *p* < 0.05 vs. siCL-CL group. #, *p* < 0.05 vs. siCL-BMP7 group. (**B**) *, *p* < 0.05 vs. siCL group. (**C**) *, *p* < 0.05 vs. 0 h group. #, *p* < 0.05 vs. siCL-24 h group. Scale bars in (**A**,**C**) are 100 μm.

Finally, we determined whether Smad5 and p75NTR are involved in regulating the BMP7 effect on human LN18 glioblastoma cell transmigration and migration. Cells were transfected with control-, Smad5- or p75NTR-specific siRNA and then kept as the controls or treated with BMP7 (50 ng/mL) for 24 h. The transmigration and migration capabilities of human LN18 glioblastoma cells were determined by the transwell and wound-healing assay. Smad5 or p75NTR gene knockdown could significantly attenuate the transmigration level of human LN18 glioblastoma cells with and without BMP7 treatment (Figure 6A). Moreover, p75NTR-specific siRNA could effectively attenuate the endogenous p75NTR expression level of human LN18 glioblastoma cells (Figure 6B and Figure S2). Furthermore, p75NTR gene knockdown could also significantly attenuate the migration level of human LN18 glioblastoma cells with and without BMP7 treatment (Figure 6C).

## 4. Discussion

**Figure 7:**
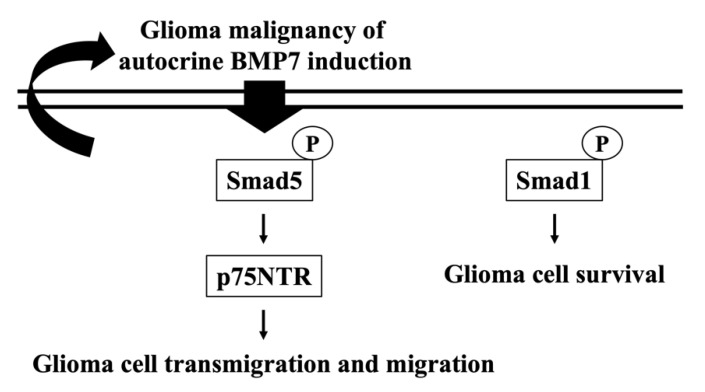
Schematic representation of the mechanism affecting autocrine BMP7 stimulated Smad5/p75NTR upregulation and subsequent glioblastoma transmigration and migration in human LN18 glioblastoma cells.

Glioblastoma is the most common malignant brain tumor with a poor prognosis. One of the major reasons for its therapy difficulty might be the presence of GICs [3,6]. GICs have high self-renewal, propagation, and tumor initiation characteristics. These lead to the relapse occurrence after treating by chemotherapeutic drugs and/or radiotherapy [1,3,5,6]. GICs have been found having the neuron stem cell-like property. Thus, the growth factors, e.g., BMP4 and BMP7, which present and play a role in the neuron differentiation and development have been suggested to be of importance for glioma recurrence and these factors should be further investigated for future pharmaceutic targets [24,25]. Recently, in addition to the tumorigenicity inhibitory activity, BMP7 has been found as another interesting role in the glioblastoma invasion promotion [13]. Based on this interesting finding, the present study further found that the endogenous and exogenous BMP7 stimulations indeed have an important role to promote the transmigration and migration capabilities of glioblastoma in human LN18/LN229 glioblastoma cells through Smad5-p75NTR pathway (summarized in Figure 7). The systematic experiments demonstrated that (i) a higher BMP7/pSmad5 level is found in the human malignant glioma tissues compared to the healthy brain tissues; (ii) BMP7 promotes human LN18/LN229 glioblastoma cell transmigration and migration through autocrine stimulation; (iii) Smad5 increases the p75NTR level to regulate the BMP7 effect on human LN18 glioblastoma cell transmigration and migration; (iv) Smad1 gene knockdown in human LN18 glioblastoma cells directly initiate their cell death.

BMPs, e.g., BMP2/4/7, and their downstream signaling, type I receptor [26,27], have been found for their potential inhibitory roles in glioma tumorigenicity since 2006 [28]. However, after extensive investigations, although most of the studies have demonstrated their glioma-suppressor roles [9,10,11,12,13,14,15], accumulating data indicated that the BMPs effect on glioma development might be tumor phenotype-dependent [24]. This is due to the fact that BMP4 and BMP7 have also been found for their invasion-promoting activities in glioblastoma cells [13,29]. Our present study further demonstrated that the transmigration- and migration-stimulating effect of BMP7 on human LN18/LN229 glioblastoma cells might be through the autocrine stimulation. Moreover, our data also accidently found the Smad1 potential role in maintaining the glioblastoma cell survival since its gene knockdown resulted in the cell death. In the clinical studies, the role of Smad1 expression and activation in the cell fate regulation of glioma is still unclear. Some data have found that the higher Smad1 activation may be beneficial to the patients’ survival [30]. However, the conflictive evidences have indicated that the patients with lower Smad1 expression have a lower survival rate or the analytic data found that the patient numbers with upregulated and downregulated Smad1 levels are almost equal [24]. Combined with the results described above, including ours, the role of Smad1 expression and activation in glioma development and in patients’ survival might be a potential candidate for future therapeutic target and/or prognostic marker and should be further examined.

Previous studies from others and us have demonstrated the central regulator role of p75NTR in glioma migration and invasion, which transduces the glioma malignant signaling through nerve growth factor-p75NTR interaction and γ-secretase cleavage [16,17,18,19,20,21,22,31]. Moreover, it has been found that BMPs and p75NTR signaling might have cross-linking in regulating the neuron development, e.g., (i) BMP7 increases p75NTR expression to induce the dendritic growth of neuron [32] and (ii) BMP9 induces p75NTR expression in basal forebrain cholinergic neurons to regulate acetylcholine synthesis [33]. In the present study, our result also elucidated that BMP7 could increase p75NTR expression to modulate glioblastoma cell transmigration and migration. Moreover, this p75NTR expression was regulated by only Smad5, not Smad1. In the neuron and glioma cell fates regulation, P75NTR is a context-dependent receptor. In addition to the binding of different ligands, including NGF and neurotrophin, its association with different types of receptors, including Trk and Sortilin, could also elicit different cell functions [16,17,18,19]. In addition, recently, the accumulating pharmaceutic study of glioma therapy is focused on the γ-secretase cleavage of p75NTR. This is due to the fact that the γ-secretase activity inhibition has been associated with the glioma invasion block [20,21]. Individual BMPs and p75NTR signaling are complex regulatory networks and impact glioma tumorigenicity and invasion. Although our present study lacks the further data to elucidate more detailed and precise mechanism between BMP7 and p75NTR cross-linking and the relationship of BMP7-Smad5 signaling and p75NTR cleavage by γ-secretase, we firmly believe that this is an important issue for future investigation.

The present study has found an autocrine BMP7 effect on glioblastoma transmigration and migration in human LN18 glioblastoma cells through Smad5, but not Smad1, and p75NTR signaling pathway. Although the results from the commercial human tissue microarray demonstrated the correlation between BMP7 level and glioma malignant stages, the limitation of the present study is that only one type of glioblastoma cells, i.e., human LN18 glioblastoma cell, is used in the present study and no further animal evidence. Moreover, our present study also suggests that the interesting data about Smad1 gene knockdown in human LN18 glioblastoma cells leading to the cell death might be an important pharmaceutic target finding for glioblastoma cell survival. All of these issues should be our future research topics and could be further elucidated.

## Data Availability

The original images of Western blot results (Figure 2, Figure 4, Figure 5 and Figure 6) are presented in Supplementary Materials-Figure S2.

## Supplementary Materials

The following are available online at https://www.mdpi.com/article/10.3390/life11070708/s1. Figure S1: IgG negative control antibody staining shows the BMP7 antibody specificity in grade 4 glioblastoma tissue; Figure S2: Gel images of Western blot.

## References

[1] Johnston A.L., Lun X., Rahn J.J., Liacini A., Wang L., Hamilton M.G., Parney I.F., Hempstead B.L., Robbins S.M., Forsyth P.A. (2007). The p75 neurotrophin receptor is a central regulator of glioma invasion. PLoS Biol..

[2] Bonavia R., Inda M.M., Cavenee W.K., Furnari F.B. (2011). Heterogeneity maintenance in glioblastoma: A social network. Cancer Res..

[3] McLendon R.E., Rich J.N. (2011). Glioblastoma Stem Cells: A Neuropathologist’s View. J. Oncol..

[4] Giese A., Bjerkvig R., Berens M.E., Westphal M. (2003). Cost of migration: Invasion of malignant gliomas and implications for treatment. J. Clin. Oncol..

[5] Giese A., Westphal M. (1996). Glioma invasion in the central nervous system. Neurosurgery.

[6] Delgado-Martín B., Medina M. (2020). Advances in the Knowledge of the Molecular Biology of Glioblastoma and Its Impact in Patient Diagnosis, Stratification, and Treatment. Adv. Sci..

[7] Katagiri T., Watabe T. (2016). Bone Morphogenetic Proteins. Cold Spring. Harb. Perspect. Biol..

[8] Davis H., Raja E., Miyazono K., Tsubakihara Y., Moustakas A. (2016). Mechanisms of action of bone morphogenetic proteins in cancer. Cytokine Growth Factor Rev..

[9] Bao Z., Zhang C., Yan W., Liu Y., Li M., Zhang W., Jiang T. (2013). BMP4, a strong better prognosis predictor, has a subtype preference and cell development association in gliomas. J. Transl. Med..

[10] Liu C., Tian G., Tu Y., Fu J., Lan C., Wu N. (2009). Expression pattern and clinical prognostic relevance of bone morphogenetic protein-2 in human gliomas. Jpn. J. Clin. Oncol..

[11] Caja L., Bellomo C., Moustakas A. (2015). Transforming growth factor β and bone morphogenetic protein actions in brain tumors. FEBS Lett..

[12] Tate C.M., Pallini R., Ricci-Vitiani L., Dowless M., Shiyanova T., D’Alessandris G.Q., Morgante L., Giannetti S., Larocca L.M., di Martino S. (2012). A BMP7 variant inhibits the tumorigenic potential of glioblastoma stem-like cells. Cell Death Differ..

[13] Savary K., Caglayan D., Caja L., Tzavlaki K., Bin Nayeem S., Bergström T., Jiang Y., Uhrbom L., Forsberg-Nilsson K., Westermark B. (2013). Snail depletes the tumorigenic potential of glioblastoma. Oncogene.

[14] Chirasani S.R., Sternjak A., Wend P., Momma S., Campos B., Herrmann I.M., Graf D., Mitsiadis T., Herold-Mende C., Besser D. (2010). Bone morphogenetic protein-7 release from endogenous neural precursor cells suppresses the tumourigenicity of stem-like glioblastoma cells. Brain.

[15] Klose A., Waerzeggers Y., Monfared P., Vukicevic S., Kaijzel E.L., Winkeler A., Wickenhauser C., Löwik C.W., Jacobs A.H. (2011). Imaging bone morphogenetic protein 7 induced cell cycle arrest in experimental gliomas. Neoplasia.

[16] Berghoff J., Jaisimha A.V., Duggan S., MacSharry J., McCarthy J.V. (2015). Gamma-secretase-independent role for cadherin-11 in neurotrophin receptor p75 (p75(NTR)) mediated glioblastoma cell migration. Mol. Cell Neurosci..

[17] Reichardt L.F. (2006). Neurotrophin-regulated signalling pathways. Philos. Trans. R. Soc. B. Biol. Sci..

[18] Roux P.P., Bhakar A.L., Kennedy T.E., Barker P.A. (2001). The p75 neurotrophin receptor activates Akt (protein kinase B) through a phosphatidylinositol 3-kinase-dependent pathway. J. Biol. Chem..

[19] Nykjaer A., Lee R., Teng K.K., Jansen P., Madsen P., Nielsen M.S., Jacobsen C., Kliemannel M., Schwarz E., Willnow T.E. (2004). Sortilin is essential for proNGF-induced neuronal cell death. Nature.

[20] Forsyth P.A., Krishna N., Lawn S., Valadez J.G., Qu X., Fenstermacher D.A., Fournier M., Potthast L., Chinnaiyan P., Gibney G.T. (2014). p75 neurotrophin receptor cleavage by α- and γ-secretases is required for neurotrophin-mediated proliferation of brain tumor-initiating cells. J. Biol. Chem..

[21] Wang L., Rahn J.J., Lun X., Sun B., Kelly J.J., Weiss S., Robbins S.M., Forsyth P.A., Senger D.L. (2008). Gamma-secretase represents a therapeutic target for the treatment of invasive glioma mediated by the p75 neurotrophin receptor. PLoS Biol..

[22] Wang T.C., Luo S.J., Lin C.L., Chang P.J., Chen M.F. (2015). Modulation of p75 neurotrophin receptor under hypoxic conditions induces migration and invasion of C6 glioma cells. Clin. Exp. Metastasis.

[23] McDonald J.W., Pilgram T.K. (1999). Nuclear expression of p53, p21 and cyclin D1 is increased in bronchioloalveolar carcinoma. Histopathology.

[24] Hover L.D., Abel T.W., Owens P. (2015). Genomic Analysis of the BMP Family in Glioblastomas. Transl. Oncogenomics.

[25] Nakano I., Saigusa K., Kornblum H.I. (2008). BMPing off glioma stem cells. Cancer Cell.

[26] Raja E., Morikawa M., Nishida J., Tanabe R., Takahashi K., Seeherman H.J., Saito N., Todo T., Miyazono K. (2019). Tyrosine kinase Eph receptor A6 sensitizes glioma-initiating cells towards bone morphogenetic protein-induced apoptosis. Cancer Sci..

[27] Raja E., Komuro A., Tanabe R., Sakai S., Ino Y., Saito N., Todo T., Morikawa M., Aburatani H., Koinuma D. (2017). Bone morphogenetic protein signaling mediated by ALK-2 and DLX2 regulates apoptosis in glioma-initiating cells. Oncogene.

[28] Piccirillo S.G., Reynolds B.A., Zanetti N., Lamorte G., Binda E., Broggi G., Brem H., Olivi A., Dimeco F., Vescovi A.L. (2006). Bone morphogenetic proteins inhibit the tumorigenic potential of human brain tumour-initiating cells. Nature.

[29] Zhou Y., Liu Y., Zhang J., Yu D., Li A., Song H., Zhang W., Davis D., Gilbert M.R., Liu F. (2020). Autocrine BMP4 Signaling Enhances Tumor Aggressiveness via Promoting Wnt/β-Catenin Signaling in IDH1-mutant Gliomas. Transl. Oncol..

[30] Liu S., Tian Z., Yin F., Zhang P.W.Y., Ding X., Wu H., Wu Y., Peng X., Yuan J., Qiang B. (2009). Expression and functional roles of Smad1 and BMPR-IB in glioma development. Cancer Investig..

[31] Yang W.H., Cheng C.Y., Chen M.F., Wang T.C. (2018). Cell Subpopulations Overexpressing p75NTR Have Tumor-initiating Properties in the C6 Glioma Cell Line. Anticancer Res..

[32] Courter L.A., Shaffo F.C., Ghogha A., Parrish D.J., Lorentz C.U., Habecker B.A., Lein P.J. (2016). BMP7-induced dendritic growth in sympathetic neurons requires p75(NTR) signaling. Dev. Neurobiol..

[33] Schnitzler A.C., Lopez-Coviella I., Blusztajn J.K. (2008). Differential modulation of nerve growth factor receptor (p75) and cholinergic gene expression in purified p75-expressing and non-expressing basal forebrain neurons by BMP9. Brain Res..

