# Supplementary material for: Bone Morphogenetic Protein 7 Effect on Human Glioblastoma Cell Transmigration and Migration
Source: Life (Basel). 2021 Jul 17;11(7):708. doi: 10.3390/life11070708 (PMC8307702; doi:10.3390/life11070708)
Supplement: Supplementary file 1 [file life-11-00708-s001.zip › Supplementary materials-final.pptx]

## Slide 1
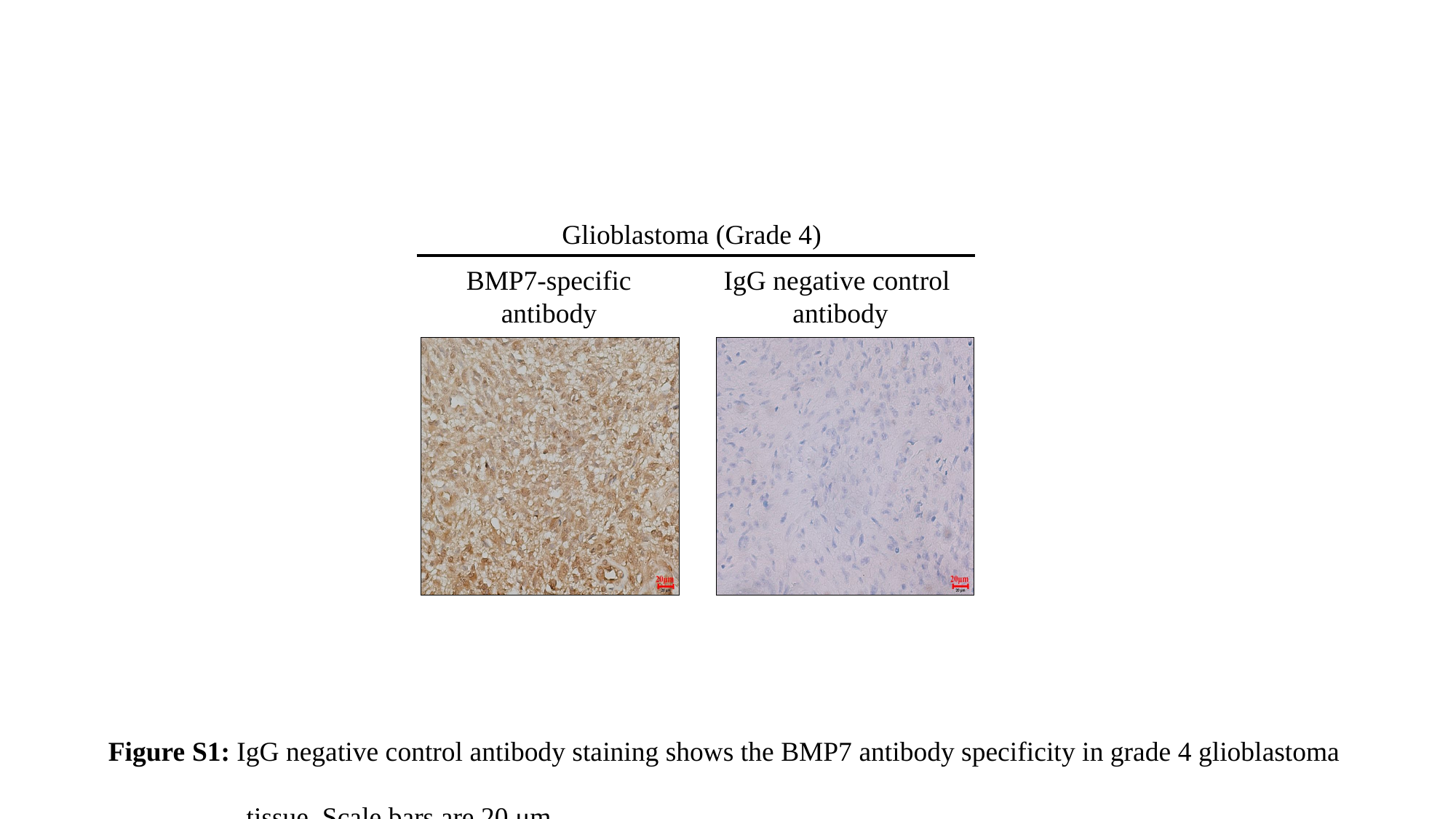

Glioblastoma (Grade 4)
BMP7-specific antibody
IgG negative control
antibody
Figure S1: IgG negative control antibody staining shows the BMP7 antibody specificity in grade 4 glioblastoma
 tissue. Scale bars are 20 μm.

## Slide 2
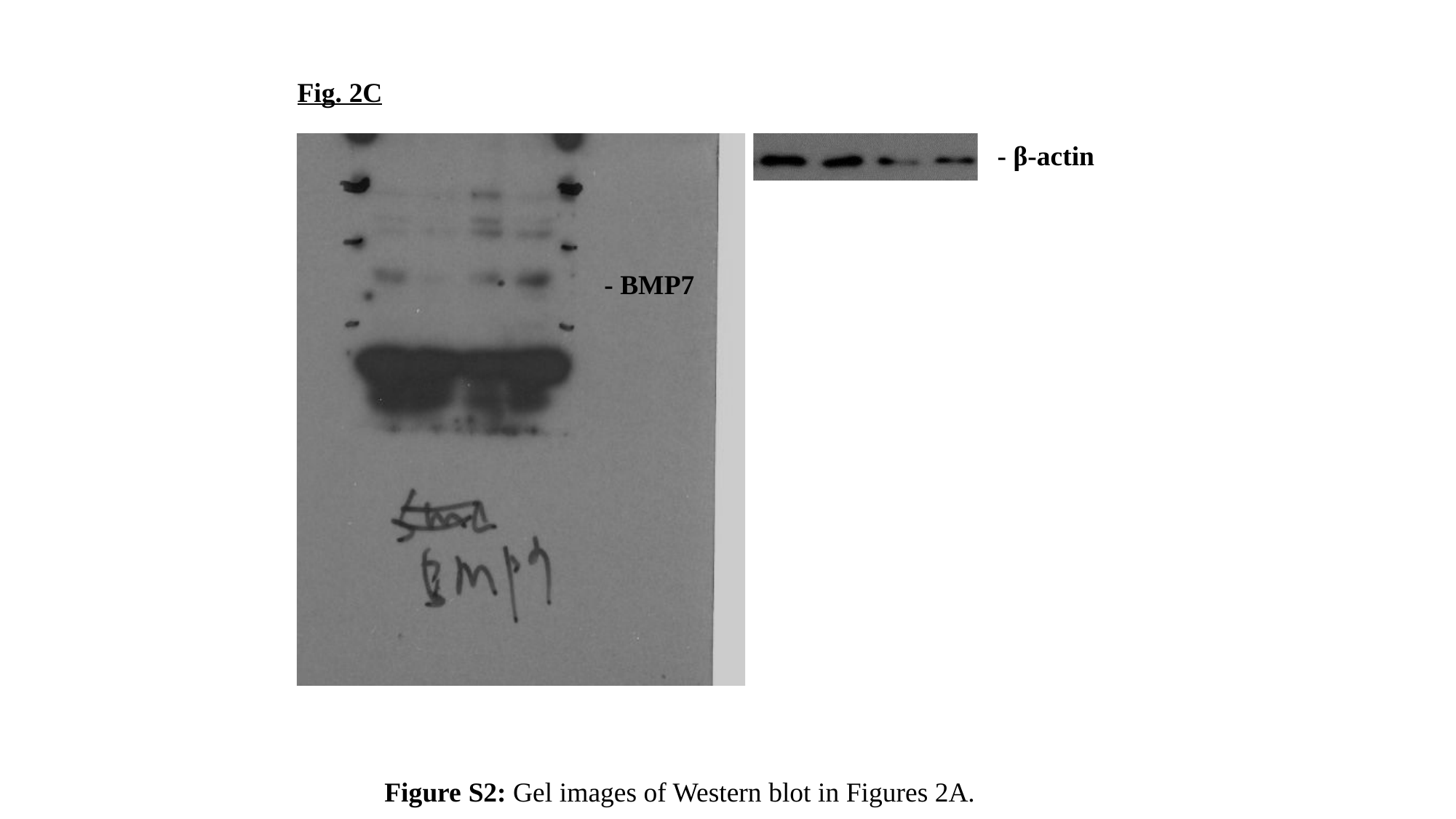

Fig. 2C
- β-actin
- BMP7
Figure S2: Gel images of Western blot in Figures 2A.

## Slide 3
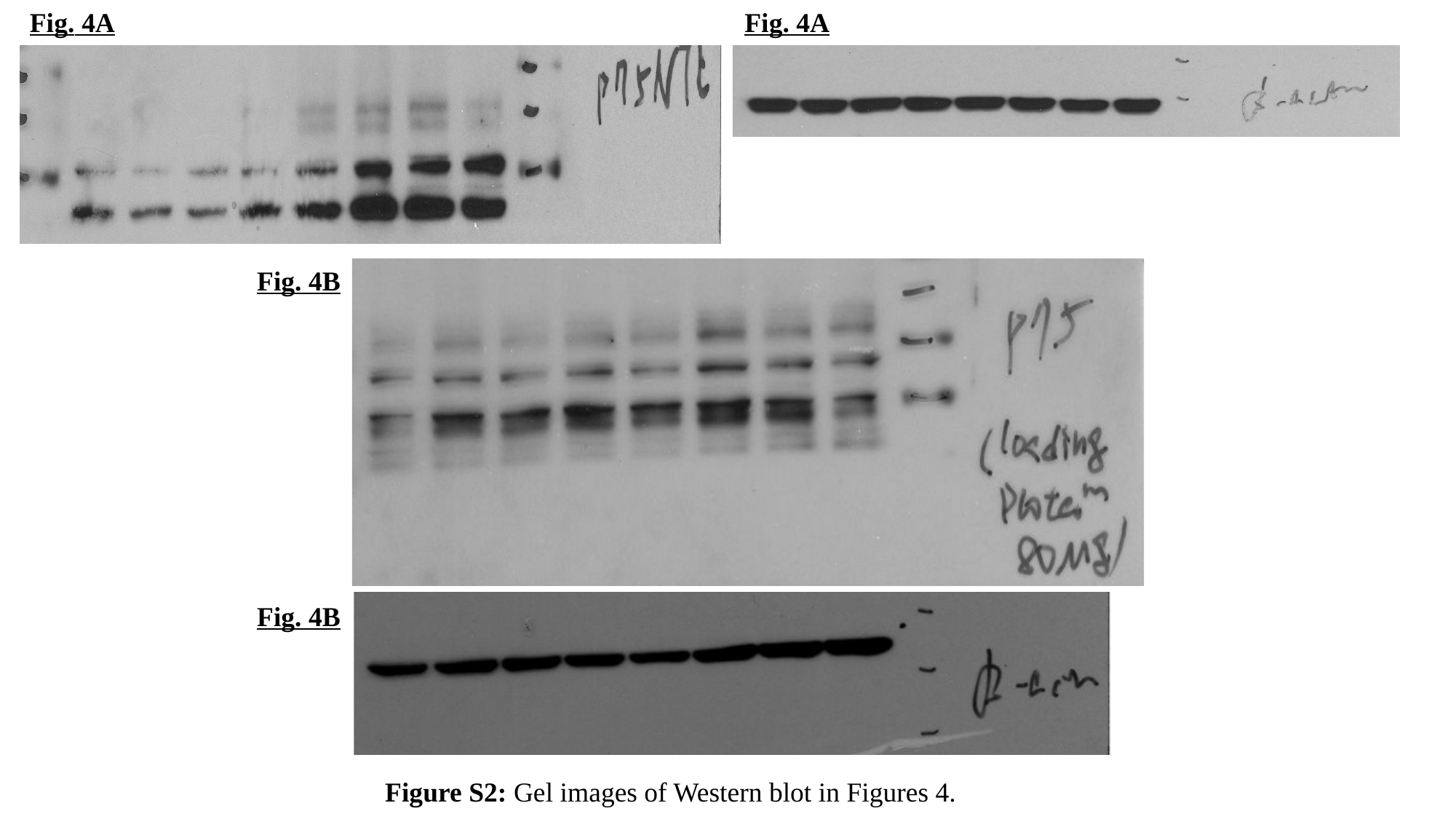

Fig. 4A
Fig. 4A
Fig. 4B
Fig. 4B
Figure S2: Gel images of Western blot in Figures 4.

## Slide 4
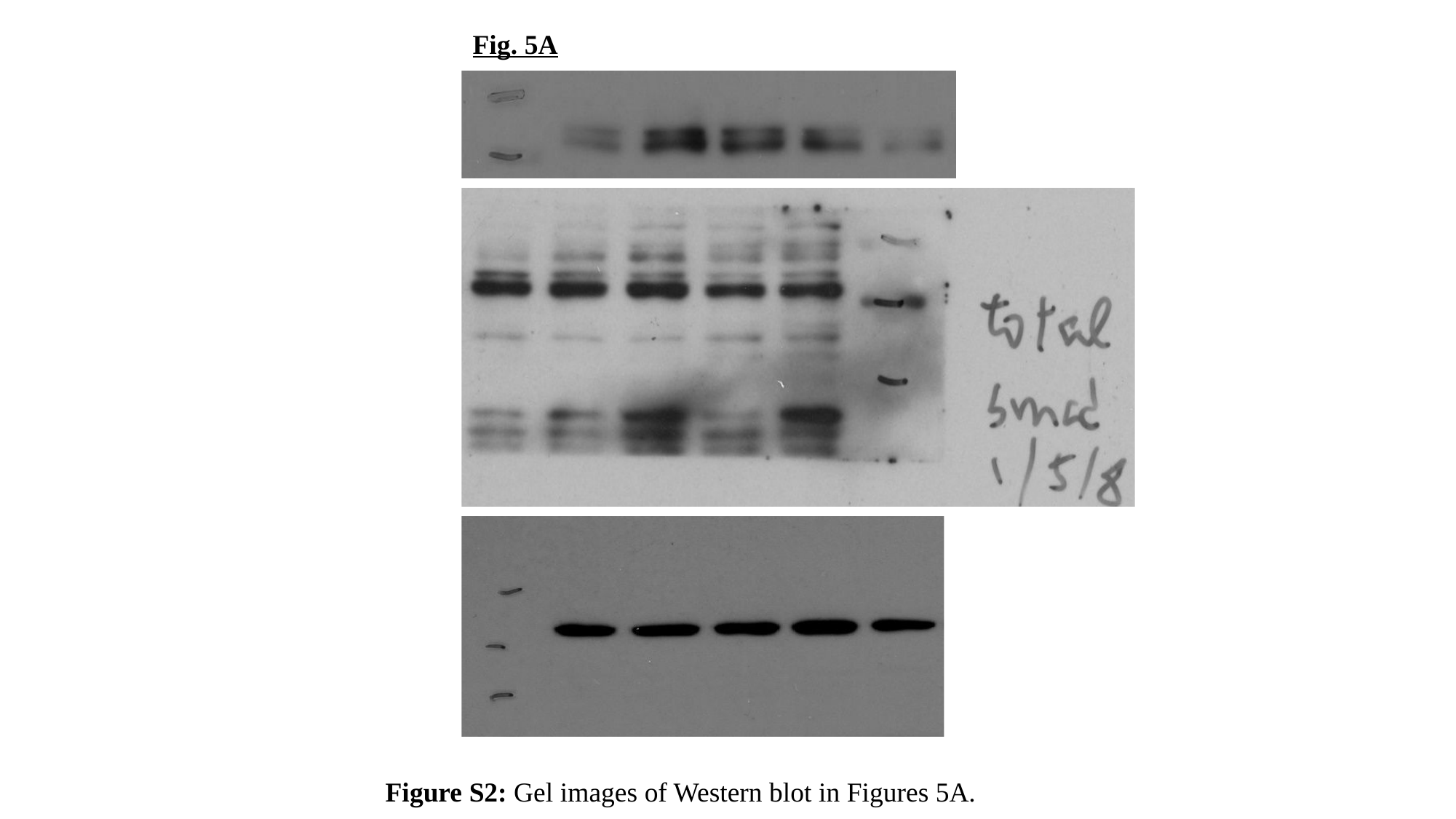

Fig. 5A
Figure S2: Gel images of Western blot in Figures 5A.

## Slide 5
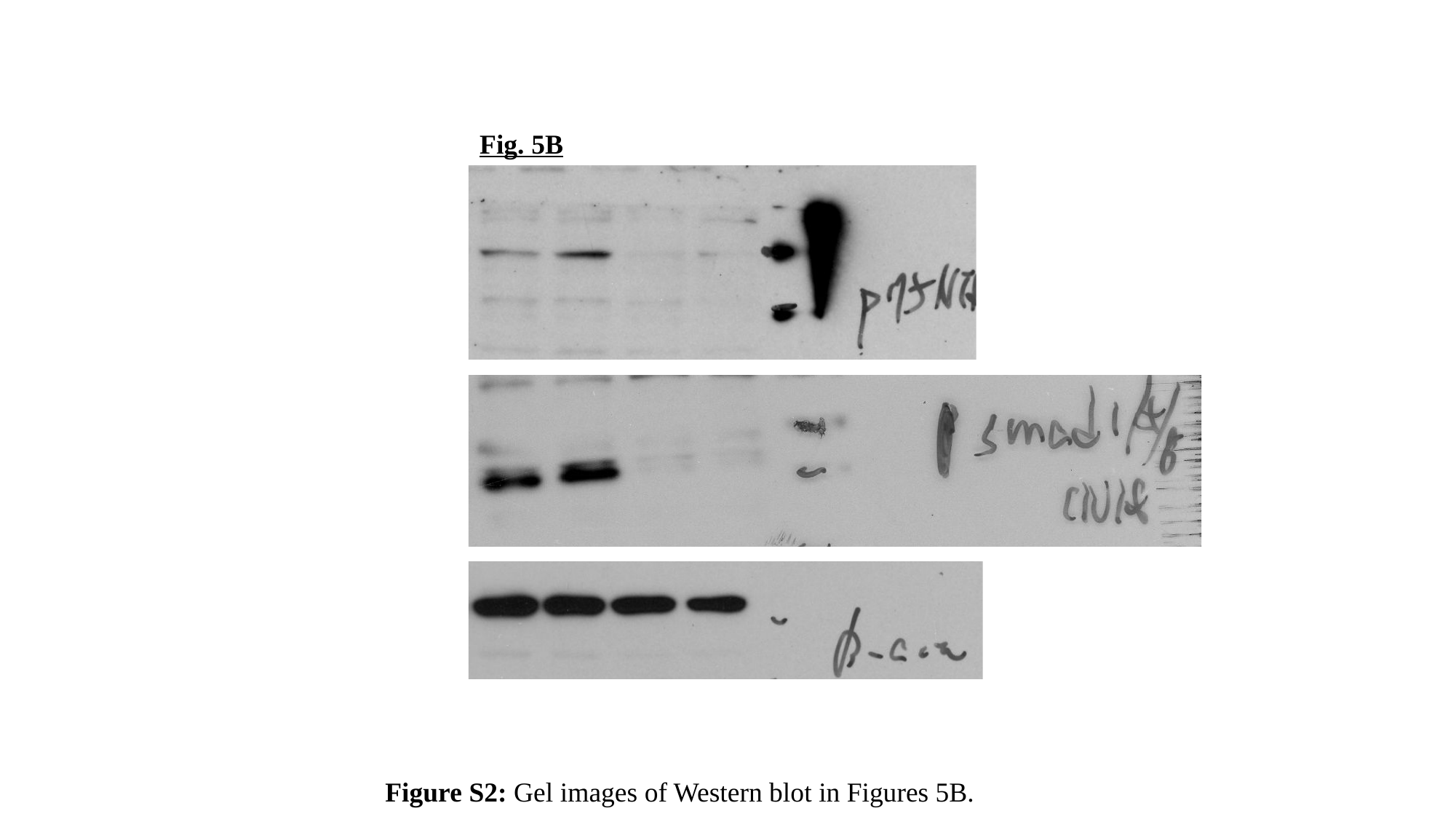

Fig. 5B
Figure S2: Gel images of Western blot in Figures 5B.

## Slide 6
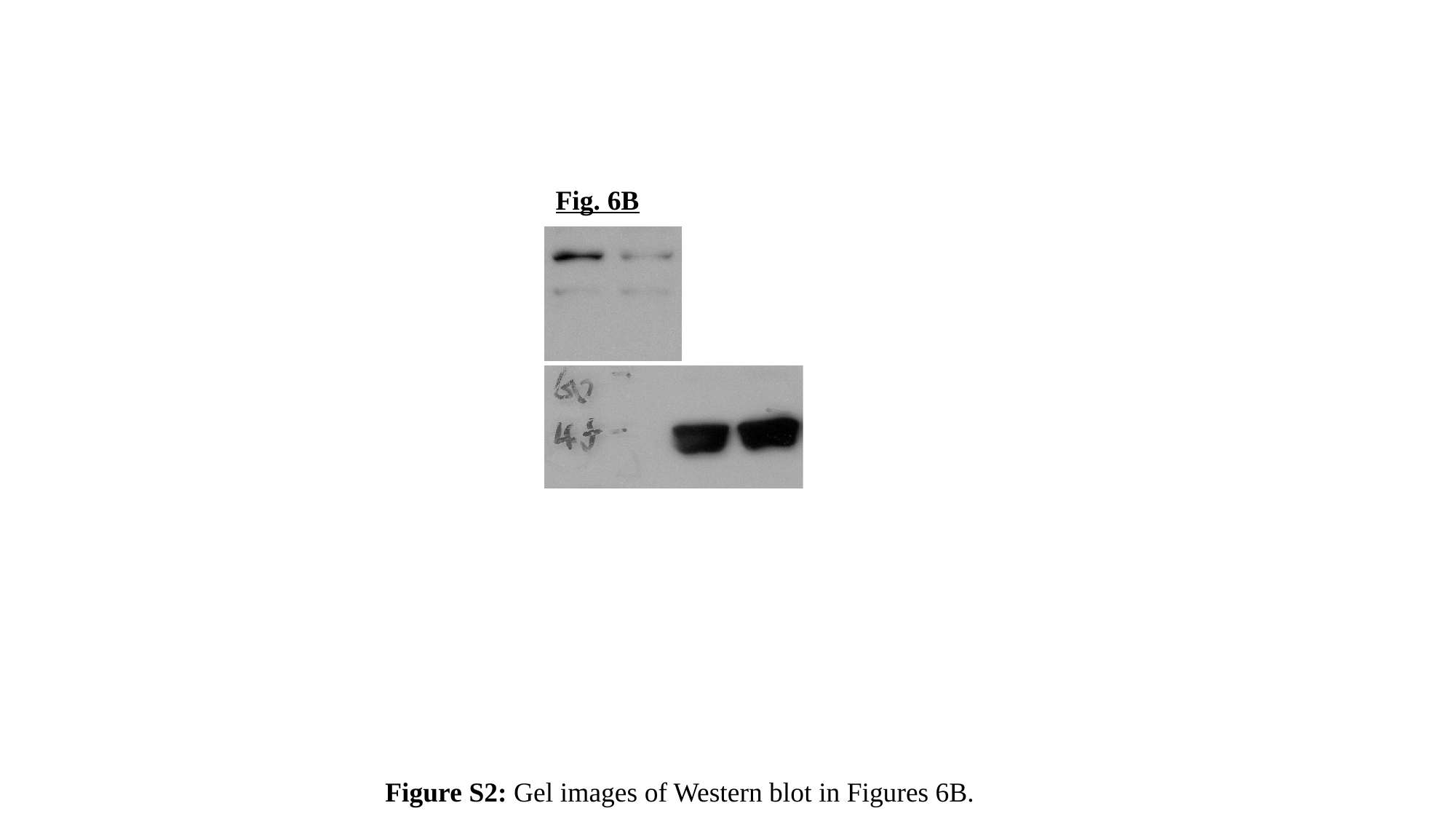

Fig. 6B
Figure S2: Gel images of Western blot in Figures 6B.
